# miR-133b, a muscle-specific microRNA, is a novel prognostic marker that participates in the progression of human colorectal cancer via regulation of CXCR4 expression

**DOI:** 10.1186/1476-4598-12-164

**Published:** 2013-12-13

**Authors:** Fang-Ting Duan, Feng Qian, Ke Fang, Kang-Yu Lin, Wen-Tao Wang, Yue-Qin Chen

**Affiliations:** 1 Key Laboratory of Gene Engineering of the Ministry of Education, State Key Laboratory for Biocontrol, Sun Yat-sen University, Guangzhou 510275, China; 2 Department of Surgery, Southwest Hospital, Third Military Medical University, Chongqing 400038, China

**Keywords:** CXCR4, miR-133b, Colorectal cancer, Tumor progression, Metastasis, Targeted therapy

## Abstract

**Background:**

MicroRNA-133b (miR-133b), which is a muscle-specific microRNA, has been reported to be downregulated in human colorectal carcinoma (CRC) when compared to adjacent non-tumor tissue. However, its diagnostic value and role in CRC have yet to be described. CXC chemokine receptor-4 (CXCR4), which participates in multiple cell processes such as cell invasion-related signaling pathways, was predicted to be a potential target of miR-133b. The aim of this study was to investigate the associations and functions of miR-133b and CXCR4 in CRC initiation and invasion.

**Methods:**

Mature miR-133b and *CXCR4* expression levels were detected in 31 tumor samples and their adjacent, non-tumor tissues from patients with CRC, as well as in 6 CRC cell lines, using real-time quantitative RT-PCR (qRT-PCR). Luciferase reporter assays and Western blots were used to validate *CXCR4* as a putative target gene of miR-133b. Regulation of *CXCR4* expression by miR-133b was assessed using qRT-PCR and Western blot analysis, and the effects of exogenous miR-133b and CXCR4 on cell invasion and migration were evaluated *in vitro* using the SW-480 and SW-620 CRC cell lines.

**Results:**

A significant downregulation of miR-133b was observed in 93.55% of CRC tissues, and the expression of miR-133b was much lower in metastatic tumors (stage C and D, stratified by the Modified Dukes Staging System) than in primary tumors (stage A and B). In contrast, CXCR4 protein expression significantly increased in 52.63% of CRC samples, and increased CXCR4 expression in CRC was associated with advanced tumor stage. *CXCR4* was shown to be a direct target of miR-133b by luciferase reporter assays, and transfection of miR-133b mimics inhibited invasion and stimulated apoptosis of SW-480 and SW-620 CRC cells.

**Conclusions:**

Our study demonstrated that downregulated miR-133b contributed to increased cell invasion and migration in CRC by negatively regulating CXCR4. These findings may be significant for the development of therapy target for CRC.

## Introduction

Colorectal cancer (CRC) is the third most common cancer in males and the second most common cancer in females worldwide and has high incidence and mortality rates [1]. The number who are affected continues to rise, especially in most Asian countries [2]. Despite gradually improved therapeutic schedules, post-operative recurrence and metastasis remain the two most challenging problems for prolonging patient survival time after surgery. Thus, it is necessary to understand the precise molecular mechanisms that modulate malignant transformation.

MicroRNAs (miRNAs), which are a class of endogenous, single-stranded RNA molecules of 20–25 nucleotides in length [3], have emerged as critical regulators of carcinogenesis and tumor progression over the last decade [4,5] and are likely to be involved in widespread biological functions, such as cell proliferation, apoptosis, invasion, angiogenesis and metastasis [5,6]. In addition, reports have increasingly shown the potential of using miRNAs as novel diagnostic markers and therapeutic targets [4,7-9].

miR-133b, which is a miRNA commonly recognized as a muscle-specific molecule, participates in myoblast differentiation [10,11] and myogenic-related diseases [12,13]. Recent studies showed that miR-133b also plays a crucial role in the malignant progression of non-muscle-related diseases [14-16] such as cancer [14-19]. For example, Bandrés *et al.*[14] revealed the deregulation of miR-133b alongside 12 deregulated miRNAs in 15 CRC cell lines and 6 paired human CRC specimens. Hu *et al.*[17] uncovered receptor tyrosine kinase MET as one target of miR-133b in CRC and demonstrated its involvement in cell proliferation and apoptosis. Another study showed that the downregulation of miR-133b in CRC tissues, when compared to adjacent non-tumor tissues, was linked to poor survival [5]. However, it remains undetermined how miR-133b functions in CRC pathogenesis and progression, especially in CRC invasion and metastasis.

The CXC chemokine receptor 4 (CXCR4) belongs to the G protein-coupled receptor (GPCRs) family [20,21]. Through a specific interaction with its ligand CXCL12 (stromal cell-derived factor-1, SDF-1) [22], CXCR4 participates in the development of primary tumors and metastases [23]. The dysregulated expression of CXCR4 was detected in several human cancers that included melanoma [24], breast [25], pancreatic [26] and CRC [24]. In particular, as a versatile factor in human CRC, CXCR4 influences aspects such as proliferation [27], migration and invasion [27,28]. Understanding the regulation network of CXCR4 would give us a deeper insight into the mechanisms underlying CRC metastasis and help in the development of new therapeutic regimens.

In this study, we found that *CXCR4* was a direct target of miR-133b in colorectal cancer. We also demonstrated that miR-133b contributed to increased cell invasion by negatively regulating *CXCR4* activity in CRC carcinogenesis and progression.

## Results

### Decreased expression of miR-133b in human CRC showed significant diagnostic potential

**Figure 1:**
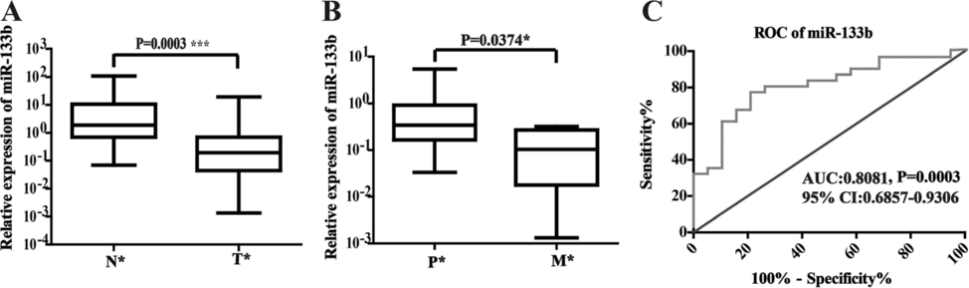
**Expression of miR-133b was downregulated in CRC. (A-B)** miR-133b expression was measured by qRT-PCR in clinical CRC tissues from 31 patients. Data were analyzed in groups, and all data were normalized to U6 snRNA. T, tumor tissue; N, adjacent normal tissues. P, primary tumors; M, metastasis tumors. **(C)** ROC of miR-133b.

To investigate whether the expression level of this muscle-specific miRNA was associated with disease progression, we first conducted qRT-PCR analyses to detect miR-133b expression in 31 human CRC tissues and their 19 counterparts from non-neoplastic adjacent tissues. As shown in Figure 1A, a significant downregulation of miR-133b was noted in 29 of the 31 tumor samples (93.55%) when compared to non-neoplastic tissues (p < 0.001), and the expression of miR-133b in metastatic tumor tissues was much lower than that in the primary tumors (p < 0.05, Figure 1B). These results implied that downregulation of miR-133b might be involved in human CRC initiation and progression.

We then examined the sensitivity and specificity of miR-133b. A receiver operating characteristic (ROC) curve analysis was performed using the relative expression of miR-133b, and the associated area under the curve (AUC) was used to confirm the diagnostic potency of the miRNA. As shown in Figure 1C, the AUC of miR-133b reached 0.8081 [95% confidence interval (CI): 0.6857-0.9306, P < 0.001], with a cut-off point of 77.42% sensitivity and 78.95% specificity. These results suggest that miR-133b can discriminate between CCA tissues and their paired adjacent normal tissues.

### *CXCR4* was a direct target of miR-133b

To gain insight into the biological role of miR-133b that underlies disease pathogenesis, we further investigated its downstream targets. Three bioinformatics algorithms, TargetScan [29], miRBase Target [30] and StarBase [31], were applied to search for the potential targets of miR-133b, and a number of potential targets were predicted. Among the predicted targets, *CXCR4* was the most interesting. *CXCR4* has been reported to be widely expressed and to exert large-scale effects in cancer cells by participating in multiple cellular processes, including cell invasion-related signaling [32]. It has also been reported that upregulation of CXCR4 was found in CRC patients and increased the risk of recurrence and poor survival from CRC [33].

**Figure 2:**
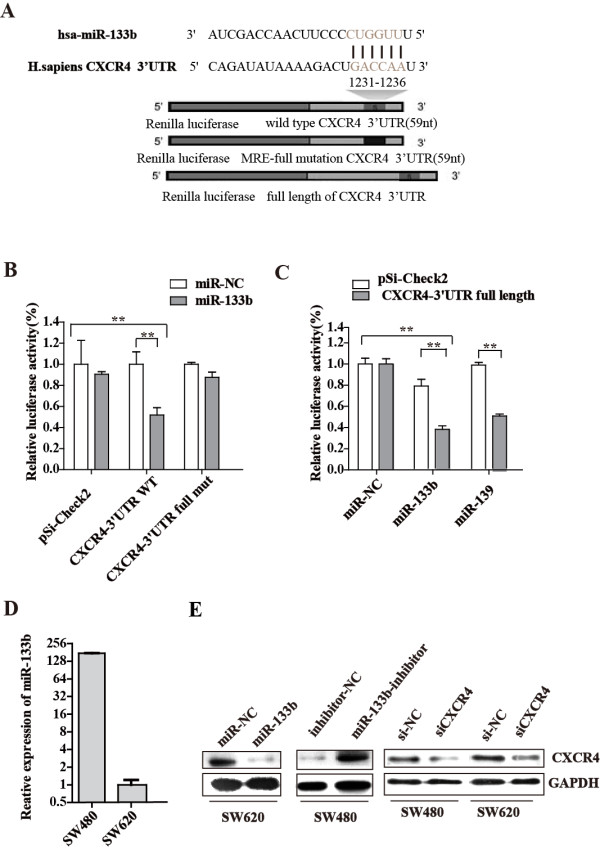
**CXCR4 is a direct target of miR-133b. (A)** Schematic of the luciferase reporter assay used to validate the interaction between miR-133b and the 3′UTR of CXCR4. Grey font indicates the ‘seed’ regions. The MRE CXCR4 3′UTR of wild, full mutant and full length were separately inserted into a psiCHECK-2 vector downstream from the *Renilla* luciferase gene. **(B-C)** Dual luciferase reporter assays performed using the 59 nt flanking the MRE, full mutation in the MRE **(B)** and the entire 3′UTR of CXCR4 **(C). (D)** miR-133b expression was measured using qRT-PCR in SW-480 and SW-620 human CRC cell lines and normalized to U6 snRNA expression. **(E)** miR-133b suppressed CXCR4 expression in CRC cells. CXCR4 protein expression of CRC cells transfected with miR-133b mimic, miR-133 inhibitor, siCXCR4 or a negative control normalized to GAPDH expression. Data are shown as the mean ± SD from three independent assays. *P < 0.05, ** P < 0.01 compared to controls.

We then validated the binding of miR-133b to the 3′UTR of *CXCR4* using a luciferase reporter assay, and miR-139 served as a positive control [34]. The detailed information used for the dual luciferase reporter assays is shown in Figure 2A. Exogenous expression of miR-133b, which was induced by introducing the miR-133b duplex into HEK-293T cells using Lipofectamine 2000, suppressed the activity of a *Renilla* luciferase construct containing the miR-133b MRE (miRNA response region) of human *CXCR4* at its 3′ end by approximately 42.7% (P < 0.01) (Figure 2B). Similarly, the activity of a luciferase construct containing the entire 3′UTR of *CXCR4* was suppressed by approximately 51.6% of the *Renilla* luciferase activity (P < 0.01) by ectopic miR-133b expression (Figure 2C). Suppression of luciferase activity was abolished when a full mismatch mutation was introduced into the miR-133b–MRE within the *CXCR4* 3′UTR (Figure 2B). These data indicated that the predicted MRE was critical for the direct and specific binding of miR-133b to the *CXCR4* mRNA.

**Figure 3:**
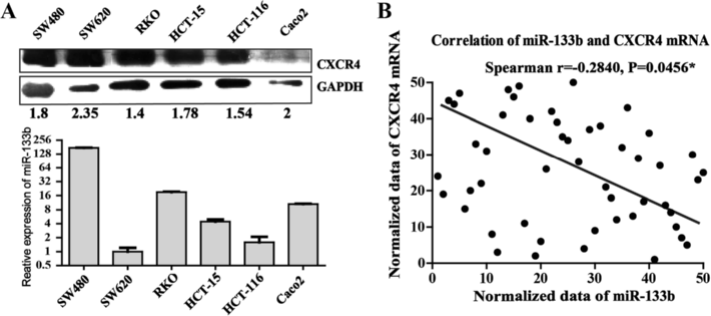
**The inverse correlation between miR-133b and CXCR4 in CRC cells and clinical samples. (A)** CXCR4 protein expression in CRC cells was detected using Western blot analysis normalized to GAPDH expression. Expression of miR-133b and the CXCR4 protein level was inversely correlated. **(B)** The inverse correlation between miR-133b and CXCR4 in CRC tissues. miR-133b and CXCR4 mRNA were measured using qRT-PCR. Correlation analysis showed a significant relationship between these factors (Spearman r = -0.2840; P = 0.0456).

To further confirm that the CXCR4 protein is suppressed by miR-133b, we then overexpressed and knocked down miR-133b in colorectal cancer cell lines. miR-133b was first detected in six CRC cell lines (SW-480, SW-620, HCT-116, HCT-15, RKO and Caco-2). Notably, the miR-133b expression level in SW-480 was considerably higher than in the other five cell lines, while the SW-620 cell line had the lowest level of expression (Figure 2D and Figure 3A). The SW-480 and SW-620 cell lines were derived from the same CRC patient but at different stages [35]. SW-480 originated from a primary tumor, and SW-620 was from a metastatic lymph node. The lower expression level of miR-133b in SW-620 than in SW-480 was consistent with the expression pattern in clinical samples. Thus, in subsequent experiments, we primarily used these two cell lines for functional studies: SW-620 was used for the gain-of-function study due to its considerably lower endogenous miR-133b level, and SW-480 was used for the loss-of-function study due to its higher level of miR-133b expression. As shown in Figure 2E, when SW-620 cells were transfected with the miR-133b mimics, the CXCR4 protein was significantly reduced. Alternatively, when the cells were transfected with the miR-133b inhibitor, CXCR4 protein expression increased in SW-480. The efficiency of siCXCR4 was verified using Western blotting (Figure 2E), and successful exogenous molecular transfection and efficiency was confirmed by qRT-PCR (Additional file 1: Figure S1A). These results indicated that *CXCR4* is a bona fide target of miR-133b.

### The inverse correlation between miR-133b and CXCR4 in CRC cell lines and clinical samples

To further validate the correlation between miR-133b and CXCR4, we then detected the expression levels of the CXCR4 protein in the six human CRC cell lines and in the clinical samples that were previously used for miR-133b detection. Intriguingly, the CXCR4 protein expression level in SW-620 was shown to be much higher than in SW-480 (Figure 3A) and was negatively coexpressed with the expression of miR-133b (Figure 3A). These results supported the hypothesis that CXCR4 is repressed by miR-133b. We then investigated the coexpression pattern between miR-133b and CXCR4 in the clinical samples. A panel of clinical samples that included CRC tissues and their corresponding adjacent non-neoplastic tissues was used.

Correlation analysis showed a significant relationship between these factors (Spearman r = -0.2840; p = 0.0456), and the results are shown in Figure 3B. The expression levels of miR-133b were significantly lower in CRC tumor tissues when compared to the NT group (Figure 1A). Conversely, the levels of CXCR4 protein were elevated in 52.63% (10/19) of the tumors when compared to their corresponding non-tumor tissues (Additional file 2: Figure S2). The remainder of the tested samples showed no significant differences between the two groups. Thus, a negative correlation exists between the level of miR-133b and the level of CXCR4 protein in CRC tumors.

### Effects of miR-133b overexpression on cell proliferation and apoptosis by modulating CXCR4 levels

To investigate whether miR-133b functions as a tumor suppressor by promoting cell apoptosis and impairing proliferation, we performed overexpression and knockdown studies to characterize the effect of miR-133b on CRC proliferation using the miR-133b mimics, miR-133b inhibitor and siCXCR4 in SW-480 and SW-620 cells. The transfection efficiencies of siCXCR4 in both cell lines are shown in Additional file 1: Figure S1. The introduction of miR-133b or knocking down CXCR4 with siCXCR4 caused a remarkable inhibition of cell proliferation in SW-480 and SW-620 cells when compared to the controls (p < 0.05 at 96 h) (Additional file 3: Figure S3A and B). In contrast, when miR-133b activity was impeded by the miR-133b inhibitor, the cells presented strengthened proliferation ability (p < 0.05 at 96 h) (Additional file 3: Figure S3C and D).

**Figure 4:**
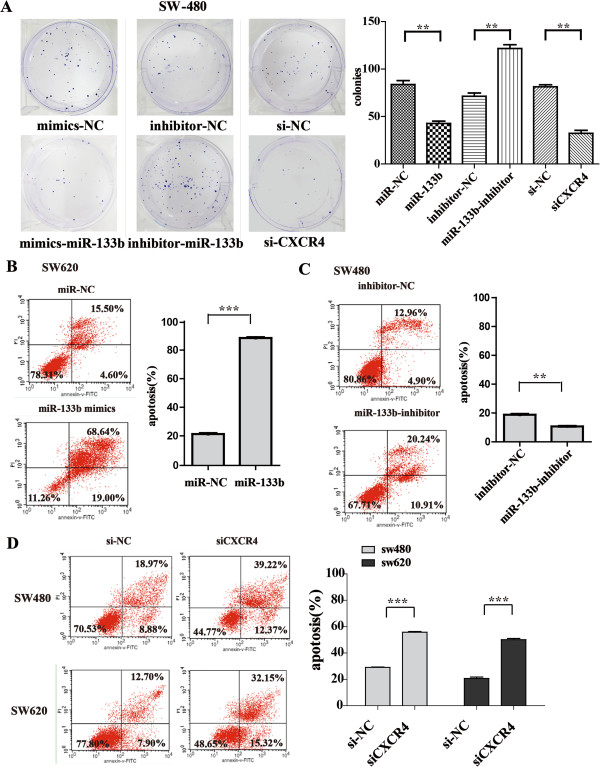
**The effect of the oligonucleotides on proliferation and apoptosis of CRC cells. (A)** Colony formation assay performed for SW-480 cells. The number of colonies on the entire plate was counted. **(B-D)** Induced apoptosis in CRC cells. After 48 h of incubation in the presence of controls or siCXCR4 and following 24-h exposure to cisplatin, all of the cells were stained with Annexin V–FITC and propidium iodide (PI) followed by flow cytometric analysis. Data are expressed as the mean ± SD of three independent experiments. **(B)** miR-133b mimic strengthened apoptosis in CRC cells. **(C)** The miR-133b inhibitor suppressed apoptosis in CRC cells. **(D)** siCXCR4 increased apoptosis in CRC cells.

A colony formation assay was performed to further substantiate this observation. The miR-133b- and siCXCR4-transfected cells formed fewer colonies than the control-transfected cells in SW-480 and SW-620 cells within 12 days, while the opposite effect was observed in cells transfected with the miR-133b inhibitor (Figure 4A and Additional file 4: Figure S4; p < 0.01). These results suggested that miR-133b could inhibit the growth of SW-480 and SW-620 cells through the targeting of CXCR4.

Proliferation and apoptosis are two classic but crucial aspects of nearly all acquired diseases. Accordingly, fluorescence-activated cell sorting (FACS) analysis was used to assess whether miR-133b contributed to apoptosis in CRC cells. Apoptosis was measured after transfecting miR-133b and siCXCR4 into SW-480 and SW-620 cells for 48 hours, and this was followed by a 24-hour exposure to cisplatin at an appropriate concentration, as previously described [36] (The IC_50_ results are not shown). The results revealed a significant increase in apoptosis of SW-620 cells transfected with miR-133b mimics compared to the control transfected cells (the percentage of apoptotic cells increased from 21.31% to 88.37%; Figure 4B; p < 0.001).

The converse effect was observed in cells transfected with miR-133b inhibitor (Figure 4C). As observed in Figure 4C, the apoptotic rate in SW-480 cells transfected with the miR-133b inhibitor dropped from 18.77% to 10.67% (p < 0.01), and this apoptosis-promoting effect of siCXCR4 was corroborated in both cell lines. In SW-480 cells, apoptosis increased from 29.13% to 55.81% (p < 0.001), and in SW-620 cells, it increased from 20.69% to 50.09% (Figure 4D, p < 0.001). The apoptosis result was further confirmed using fluorescence microscopy, in which the pretreatment of cells was similar to that by flow cytometry analysis (Additional file 5: Figure S5). These results indicate that overexpression of miR-133b induced an aggravated apoptosis rate and an impaired proliferation of CRC cells.

### Forced expression of exogenous miR-133b decreases CRC cell invasion and migration *in vitro*

**Figure 5:**
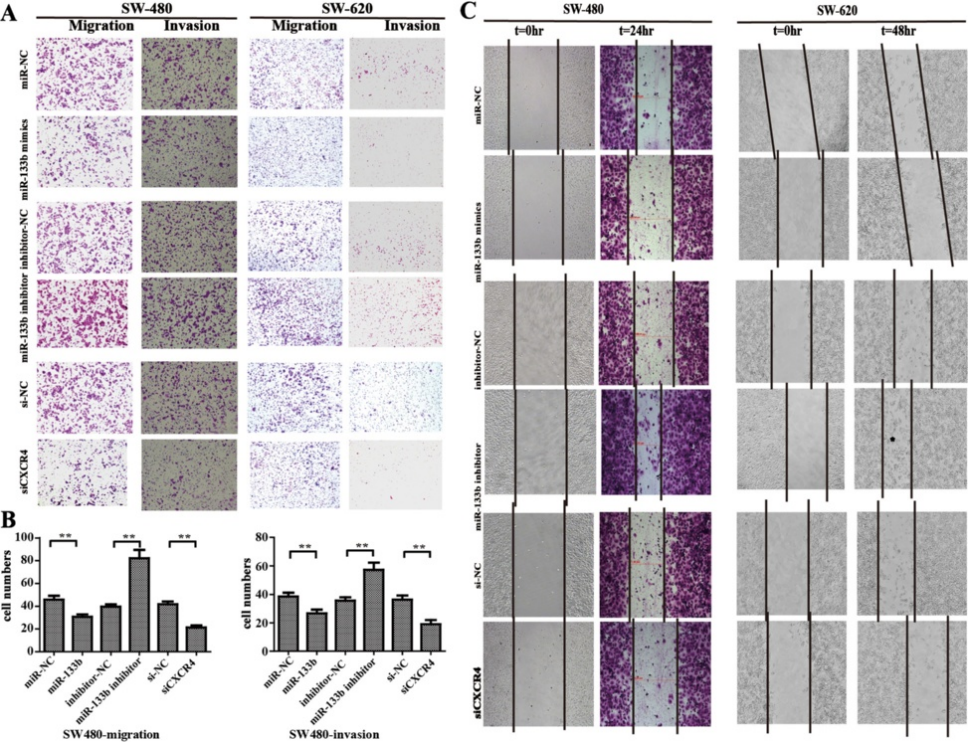
**miR-133b regulates motility of CRC cells. (A)** The transwell invasion and a migration assays were used to detect the motility of SW-480 and SW-620 cells transfected with miR-133b mimic, miR-133b inhibitor, siCXCR4 or their corresponding negative controls. **(B)** The cells that invaded or migrated to the lower upside were counted using a microscope. Original magnification: 200×. **(C)** Using a wound healing assay, the cell motilities of SW-480 and SW-620 cells transfected with the miR-133b mimic, miR-133b inhibitor, siCXCR4 or their corresponding negative controls were observed at 0, 24 and 48 hours following wounding by a pipette tip. Original magnification: 100×.

The lower expression level of miR-133b in advanced CRC SW-620 cells implied that miR-133b might contribute to the metastatic features of CRC. We postulated that ectopic expression of miR-133b in CRC cells could impede the migratory and invasive abilities of CRC cells. To confirm this speculation, miR-133b mimics were transiently introduced into the cells for 36 hours. The cells were then starved for 12 hours, and the migration assays were performed. As expected, exogenous expression of miR-133b and siCXCR4 substantially impeded the migratory ability of CRC cells, as indicated by the decreased number of migrated cells (Figure 5A). A similar result was also observed using the cell invasion assay that was counted using a microscope (Figure 5B). We also transiently transfected miR-133b inhibitors into the cells. As shown in Figure 5A and 5B, inhibition of miR-133b significantly increased cell migration and invasion, especially in SW-480 cells, which had relatively higher endogenous miR-133b expression.

**Figure 6:**
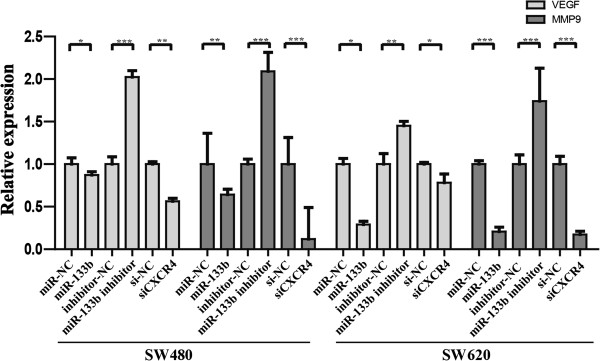
**qRT-PCR was used to detect the expression of the *VEGF *** **and *MMP-9 *** **genes in the SW-480 and SW-620 cell lines transfected with the miR-133b mimics, miR-133b inhibitor or CXCR4 siRNA.**

We used a scratch wound healing assay to further demonstrate the function of miR-133b in migration potency. Treatment with the miR-133b mimic and siCXCR4 inhibited wound closure in both cell lines compared to the control (Figure 5C). In contrast, when transfected with the miR-133b inhibitor, the speed of wound closure was increased. Our results suggest that miR-133b suppresses CRC metastasis by regulating the migratory and invasive abilities of CRC cells through CXCR4. To further reveal the potential signaling pathway that underlies the miR-133b/CXCR4 interaction, we investigate the expression of the CXCR4 downstream genes *vascular endothelial growth factor (VEGF)* and *matrix metalloproteinase-9 (MMP-9)*[37,38]. The results showed that their expressions were affected by the miR-133b mimics and inhibitor in the SW-480 and SW-620 cell lines (Figure 6), that miR-133b regulates CXCR4 to affect its classic underlying pathway.

## Discussion

CRC is one of the most common and lethal cancers and has a high relapse rate. Therefore, there is a strong need to develop novel, prognostic factors and therapeutic strategies. The outcome of CRC patients is determined primarily by the presence or absence of metastases. Thus, insight into the molecular mechanisms underlying the precise molecular mechanisms that modulate malignant transformation is required. Previous studies have shown that aberrant expression of miR-133b was found in CRC cancer tissues [14,17] and that overexpression of miR-133b induced apoptosis and G1 cell-cycle arrest in CRC cells [17]. Furthermore, miR-133b has reportedly been shown to be involved in the invasion of several other cancers. For instance, miR-133b was found to be downregulated in non-small cell lung cancer and modulate apoptosis and invasion [16], and overexpression of miR-133b has been shown to inhibit cell invasion activity in esophageal squamous cell carcinoma [19]. However, the relationship between miR-133b expression and cell metastases in CRC has yet to be demonstrated.

In the present study, we investigated the expression patterns of miR-133b in CRC clinical samples and identified low miR-133b expression as a valid factor associated with advanced tumor stages. Further functional analysis revealed the involvement of miR-133b in the progression of human CRC, and transfection of miR-133b into two CRC cell lines, SW-480 and SW-620, significantly decreased tumor cell migration and invasion *in vitro*. These data provide the potential of miR-133b to serve as a molecular target for CRC therapy, especially for tumors with high degrees of metastasis. It is also worth noting that the outcome of CRC patients is highly relevant to the extent of local invasion; therefore, the metastases-related miR-133b might provide tumor progression and prognostic information in CRC patients who would need to be experimentally validated prospectively.

We revealed the involvement of miR-133b in the progression of human CRC via the regulation of CXCR4 expression. A significant correlation was also found between miR-133b and CXCR4 protein expression in tumor samples. The activation of CXCR4, a G protein-coupled receptor for CXCL12, induced tumor invasion and/or survival of cancer cells. CXCR4 has also been reported to be involved in a number of processes related to the immune system [39], the nervous system [40], angiogenesis [41], the hemopoietic system [42] and carcinogenesis [28,43-46]. Therefore, it is a key receptor in the crosstalk between tumor cells and their microenvironment.

Our results demonstrated that the miR-133b/CXCR4 pair is involved in tumor growth and tumor cell apoptosis and controls cell migration and invasion. Intriguingly, CXCR4 has been regarded as an impressive anticancer target that suppresses the outgrowth of metastases in CRC [28]. Moreover, previous reports have shown that the small non-peptide CXCR4 inhibitor ADM3100 effectively inhibited the invasion and metastasis activity of CRC [47], which strongly shows the potential of CXCR4 as a therapy target. Furthermore, we found that the miR-133b/CXCR4 interaction influenced CRC progression through modifying the *VEGF* and *MMP-9* genes, both of which play significant roles in CRC, especially in migration and invasion [48,49]. More importantly, we determined the downstream molecules of the miR-133b/CXCR4 interaction as was done in previous research on CXCR4 in CRC [37]. This finding implies that miR-133b regulates CXCR4 to affect its classic underlying pathway, which highlights the potential of this miRNA to be used as a CXCR4 inhibitor in CRC treatment. Taken together, our research provides an alternative strategy for developing miRNA-based therapy via CXCR4 targeting in CRC, and this is considered more security for the natural and endogenous of miRNAs.

In conclusion, our current findings provide the first glimpse of the functional role of miR-133b in CRC carcinogenesis and progression through the negative regulation of CXCR4. We also identified the crucial role of this miRNA in tumor cell invasion. These results indicate that miR-133b may be a useful therapeutic target in CRC.

## Materials and methods

### Patients, tissues, cell lines and cultures

Thirty-one fresh, human CRC tissues and nineteen adjacent, non-tumor tissue counterparts (NTs) were obtained from CRC patients at the time of surgery at the Southwest Hospital Affiliated Third Military Medical University. The tumor identity was verified by pathologists. All specimens were snap-frozen in liquid nitrogen immediately after surgery and then stored at -80°C until use. Detailed clinical information for these patients is presented in Table 1. Tumors were stratified according to the internationally accepted Modified Dukes Staging System, and the study was approved by the local ethics committee. Written, informed consent was obtained from all patients.

**Table 1:** Detailed clinical information of CRC patients used in this study

	**Gender (M/F)**	**Age at diagnosis**	**Dukes classification**	**Metastasis situation**
1	F^*^	42y	C^*^	-^*^
2	F	40y	B^*^	-
3	M^*^	51y	B	-
4	M	63y	C	LN^*^
5	M	62y	C	LN
6	M	69y	C	-
7	M	33y	C	-
8	F	64y	C	-
9	F	83y	C	LN
10	M	60y	C	-
11	F	68y	A^*^	-
12	F	60y	C	-
13	M	48y	C	LN
14	M	74y	C	-
15	F	68y	B	-
16	M	68y	C	-
17	M	69y	C	LN
18	M	72y	D^*^	G^*^
19	M	65y	B	-
20	M	55y	B	-
21	F	61y	B	-
22	M	70y	B	-
23	F	85y	A	-
24	M	67y	C	LN
25	M	56y	D	L
26	M	56y	D	LN/L
27	M	65y	D	L
28	F	85y	A	-
29	M	65y	C	LN
30	M	55y	C	LN
31	M	42y	D	L

^*^F: female; M: male; A, B, C, D: Dukes A, B, C, D; -: no metastasis found; LN: lymph node metastasis; L: liver metastasis.

The HEK-293T and human CRC cell lines SW-480, SW-620, HCT-15, HCT-116, Caco-2 and RKO were purchased from the Cell Bank of the Chinese Academy of Sciences (Shanghai, China), maintained in a 37°C humidified incubator, and cultured in appropriate media as recommended by the supplier.

### Plasmid construction

Wild-type and full-mutated miR-133b putative target segments comprising 59 bp of the 3′UTR (untranslated terminal region) of CXCR4 were synthesized by Invitrogen (Invitrogen, China) and cloned into the psiCHECK-2-CXCR4 vector (Promega, Madison, WI, USA) for miRNA functional analysis. These plasmids were designated psiCHECK-2-CXCR4 wt and psiCHECK-2-CXCR4 full mut, respectively. The psiCHECK-2-CXCR4 full-mutated vector introduced the full mutation into the miR-133b binding sites of the *CXCR4* 3′UTR. Additionally, we generated a luciferase vector containing the full length 3′UTR of *CXCR4* by RT-PCR, and this was designated as psiCHECK-2-CXCR4 full length. Proper insertion was confirmed by sequencing, and all utilized primers are described in Additional file 6: Table S1.

### Cell transfection

The following oligonucleotides were purchased from GenePharma (GenePharma, Shanghai, China): miR-133b mimics; miRNA negative control (designated as miR-NC); miR-139 mimic as a positive control; miR-133b antisense with a sequence complementary to the mature miR-133b; and miRNA antisense negative control (designated as inhibitor-NC), which is a negative control for miR-133b antisense. The small interfering RNAs (siRNA) against the human CXCR4 (GenBank Access. NM_001008540.1 and NM_003467.2) transcripts (denoted as siCXCR4) and the negative control RNA duplex (denoted siRNA NC) were purchased from Guangzhou Ribo-Bio Co., Ltd (Guangzhou, China). The sequence of siCXCR4 is described in Additional file 6: Table S1. Lipofectamine 2000 (Invitrogen Corporation, Carlsbad, CA, USA) was used for reverse transfection of the small molecules as well as cotransfection of the miRNA mimics and reporter vectors at optimized concentrations (10–200 nM) according to the manufacturer’s recommendation. The plasmid pcDNA-6.2 containing GFP was used as a positive control for plasmid transfection, and Block it™ tagged with fluorescein was used as a positive control for oligonucleotide transfection. Twenty-four to 60 hours after transfection, the cells were harvested for the dual luciferase reporter assay, protein analysis or RNA extraction.

### Luciferase target assays

Once 70-80% confluent in 48-well plates, HEK-293T cells were cotransfected with 50 ng/well of each luciferase reporter plasmid and 10 nM/well of either miR-133b mimic, miR-139 mimic or miR-NC, as described above. The lysates were collected 36 hours posttransfection to determine firefly and *Renilla* luciferase activity using the Dual-Luciferase Assay Kit (Promega, Madison, WI) following the manufacturer’s instructions. All experiments were performed in triplicate.

### Total RNA extraction and quantitative reverse transcription-PCR (qRT-PCR) analysis

Total RNA was extracted from cells using TRIzol reagent (Invitrogen, Carlsbad, CA, USA) following the manufacturer’s instructions. Tissue was porphyrized in liquid nitrogen, and then the RNA was extracted with TRIzol.

The expression of mature miR-133b was determined using the Hairpin-it™ Assay kit (GenePharma, Shanghai, China) and normalized to U6-snRNA. A qRT-PCR for the CXCR4-mRNA was performed using the SYBR Premix ExTaq real-time PCR kit (Takara, Japan) according to the manufacturer’s instructions with GAPDH as the normalization controls, respectively. Each reaction was carried out in triplicate. To calculate the relative expression levels, we used the 2^-∆∆CT^-method. All primer sequences can be observed in Additional file 6: Table S1.

### Protein extraction, western blotting and antibodies

Specimens were preprocessed as mentioned above, and total protein was extracted using TRIzol reagent (Invitrogen, Carlsbad, CA, USA) as recommended. Protein samples were lysed in buffer containing 1% DTT, 4% CHAPS, 7 M urea, 2 M thiourea and 2% ampholine. A volume of extract equivalent to 15 μg of total protein was separated in a 12% SDS-PAGE gel and then transferred to a methanol-activated PVDF membrane (Millipore, Beijing, China). The membranes were blocked with 5% BSA (bovine serum albumin, Sangon, Shanghai, China) and then incubated with primary antibody that selectively recognized CXCR4 (ab2074, Abcam, USA) at 4°C overnight. To determine the amounts of loaded proteins, membranes were also blotted with anti-GAPDH antibody (Proteintech Technology, Manchester, UK). Subsequently, we incubated the membranes with HRP-conjugated secondary anti mouse (Pierce) or rabbit (Sigma-Aldrich) antibody, and then protein bands were visualized by adding ECL Plus Western blotting detection reagents (Millipore) and exposure to Kodak film following the manufacturer’s instructions. Protein levels were normalized to GAPDH.

### Cell proliferation and colony formation assays

Cell proliferation was assessed using the Cell Counting Kit-8 (CCK-8, Dojindo Molecular Technologies, Shanghai, China) as previously described. Cells were seeded as 5 replicates at a density of 6000/well in 100 μl of full medium in 96-well plates and transfected with miR-133b mimics (100 nM), miR-NC (100 nM), miR-133b inhibitor (200 nM), inhibitor-NC (200 nM), siCXCR4 (120 nM) or si-NC (120 nM) as described above. The cells were then incubated at 37°C, and the absorbance was measured at wavelengths of 480 nm and 630 nm on consecutive days for four days.

For colony formation assays, 1000 cells that had been transfected with oligonucleotides were suspended in 2 ml of full medium and then seeded in 6-well plates. The cells were washed with phosphate-buffered saline (PBS), fixed with methanol and stained with crystal violet (0.1% crystal violet in 20% methanol) after 12-day incubation. Colonies with more than 50 cells were counted, and five fields were counted for each plate. The assay was performed in triplicate for each cell line.

### Apoptosis assay (fluorescence-activated cell sorting (FACS) analysis)

Cells were transfected with the small molecules for 48 hours followed by a 24-hour exposure to cisplatin at final concentrations of 2.5 μg/ml and 1 μg/ml, respectively. After trypsinization and washing with ice-cold PBS, the cell suspensions were stained using Annexin V/FITC and propidium iodide (PI) (Annexin V/PI Apoptosis Detection Kit, Lianke, China) and then analyzed by measuring the membrane redistribution of phosphatidylserine by flow cytometry (Beckman Coulter, USA). The experiments were performed in triplicate.

### Cell migration and invasion assays

Migration and invasion assays were conducted using Transwell chambers (8 μm, Corning Costar Co., Cambridge, MA) according to the manufacturer’s instructions. Briefly, 24 hours after transfection, the cells were starved for 12 hours and then trypsinized and resuspended in serum-free medium to a final concentration of 2 × 10^5^/ml (for the migration assays) or 4 × 10^5^/ml (for the invasion assays). The cell suspension (200 μl) was then pipetted into the top chamber. Medium (600 μl) with 10% fetal bovine serum was added to the lower chamber as a chemoattractant. After 36-hour incubation, the cells on the upper side of the membrane were mechanically removed with cotton swabs, and cells that migrated to the lower surface were fixed with 100% methanol and stained with 0.1% crystal violet. The cells were counted in five fields for triplicate membranes at 10× magnification using a microscope (Zeiss).

Cell invasion assays were performed as described for the cell migration assay, but polycarbonate membranes coated with 45 μl of 300 μg/ml extracellular matrix (Matrigel; BD Biosciences, San Jose, CA) that was diluted with medium lacking FBS were used.

### Scratch wound healing assay

Transfected SW-480 and SW-620 cells were cultured in 24-well plates for 24–48 hours in standard conditions until 70-80% confluency. Linear wound tracks were generated with sterile, 10-μl pipettes and maintained under standard conditions. The scratched cells were then rinsed twice with PBS to remove non-adherent cells, and fresh culture medium was added. Photographs of the centers of the gaps were taken using a phase-contrast microscope and the same magnification, 100×. The cell migration at 0, 24, and 48 h after scratching was evaluated by determining the wound distance at two random wound gap locations. Three independent scratch-wound experiments were used for calculations.

### Statistical analysis

All statistical calculations were performed using GraphPad Prism (GraphPad Prism Software, Version 5.0, GraphPad, San Diego, CA) and SPSS (Statistical Package for the Social Scienes) PASW Statistics software (versions 17.0, USA).

Fisher’s exact test and the Mann–Whitney *U*-test were used to compare differences between two groups. The related clinical data after logarithmic transformation were used to analyze the diagnostic utility by receiver operating characteristic (ROC) curves. Discriminant analysis was conducted to find and build a model of predicted probability. The correlation between miR-133b and CXCR4 was determined by the Spearman rank correlation test. Youden’s index was used to predict the optimal cutoff point. The other data in each group were defined as the mean ± SD. All p values were two-tailed, and p < 0.05 was considered statistically significant.

## Competing interests

The authors declare that they have no competing interests.

## Authors’ contributions

FTD and FQ designed and performed the research, analyzed data and wrote the manuscript. KF, KYL and WTW performed the research and analyzed data. YQC designed the research and wrote the manuscript. All authors read and approved the final manuscript.

## Supplementary Material

Additional file 1: Figure S1 Successful exogenous molecules transfection was confirmed by qRT-PCR normalized to GAPDH/U6 snRNA expression. Data are shown as the mean ± SD from three independent assays. *P < 0.05 as compared with control. Click here for file

Additional file 2: Figure S2 Expression of CXCR4 protein in 19 paired CRC tissues was detected using a Western blot analysis normalized to GAPDH in CRC samples. In 10 of 19 patients, the expression of miR-133b in the tumors is higher than in the adjacent non-tumor tissues (signed by star). Click here for file

Additional file 3: Figure S3 The effect of miR-133b on CRC proliferation. Click here for file

Additional file 4: Figure S4 Colony formation assay performed in SW-620. The number of colonies was counted under a microscope using an original magnification of 100× to adjust for the different density of each cell line. Click here for file

Additional file 5: Figure S5 **(A)** Observation of the apoptosis of SW-480 cells transfected with small molecules under a fluorescent microscopy. **(B)** Observation of the apoptosis of SW-620 cells transfected with small molecules under a microscopy. Original magnification: 200×. Click here for file

Additional file 6: Table S1 Primers and siRNA sequences used in this study. Click here for file
