# Supplementary figures and images for: miR-133b, a muscle-specific microRNA, is a novel prognostic marker that participates in the progression of human colorectal cancer via regulation of CXCR4 expression
Source: Mol Cancer. 2013 Dec 13;12:164. doi: 10.1186/1476-4598-12-164 (PMC3866930; doi:10.1186/1476-4598-12-164)

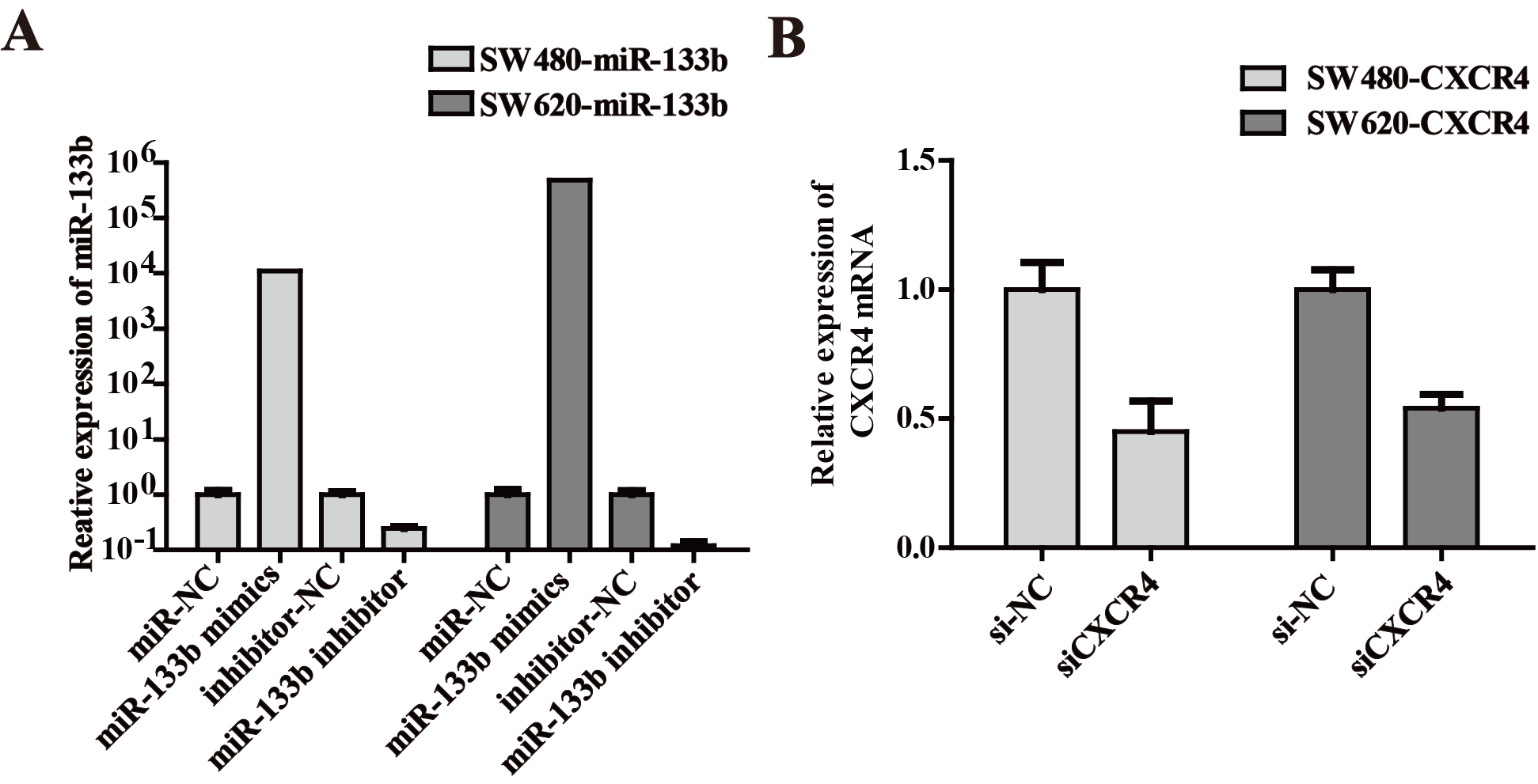

Supplement: Additional file 1: Figure S1 — Successful exogenous molecules transfection was confirmed by qRT-PCR normalized to GAPDH/U6 snRNA expression. Data are shown as the mean ± SD from three independent assays. *P < 0.05 as compared with control. [file 1476-4598-12-164-S1.tiff]

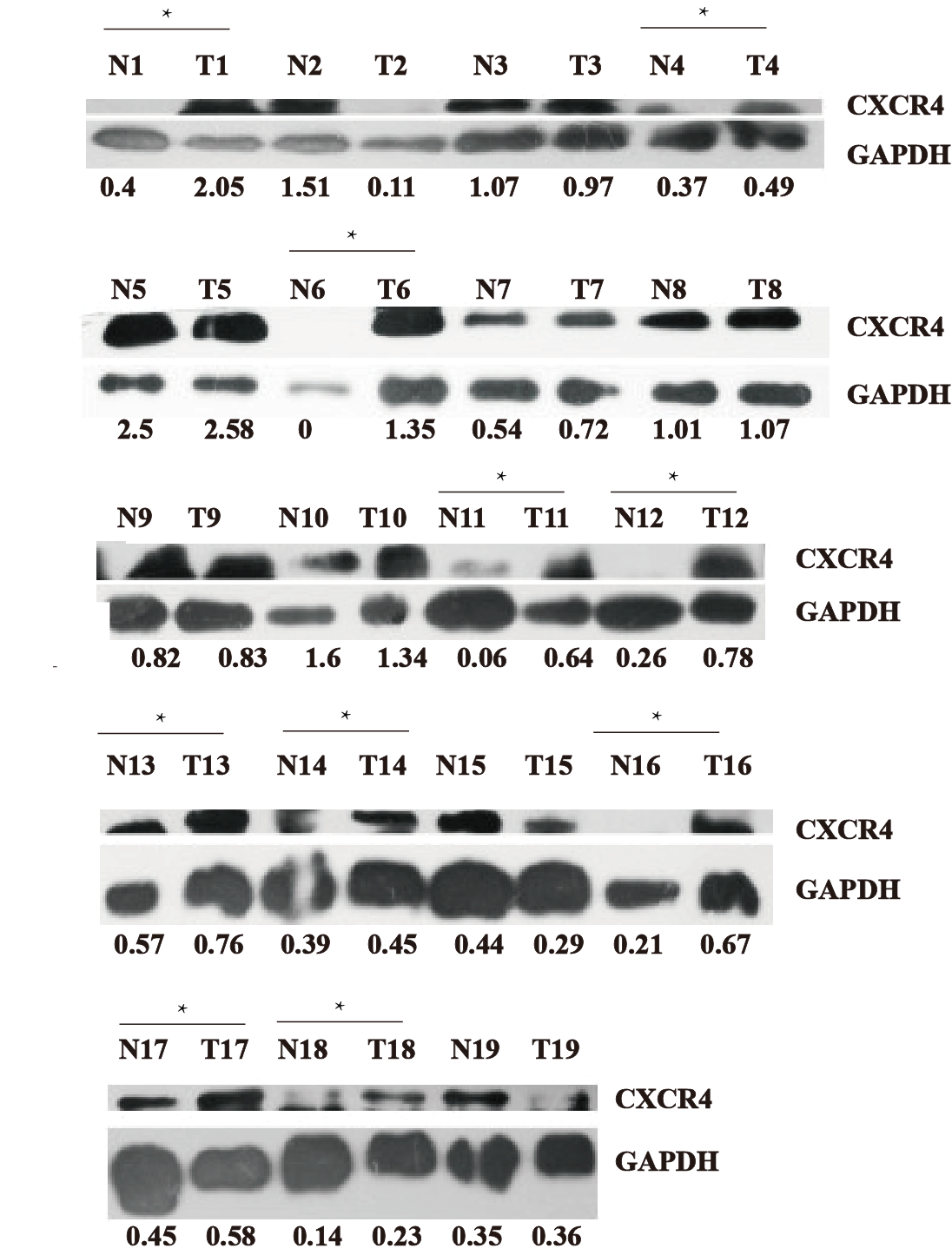

Supplement: Additional file 2: Figure S2 — Expression of CXCR4 protein in 19 paired CRC tissues was detected using a Western blot analysis normalized to GAPDH in CRC samples. In 10 of 19 patients, the expression of miR-133b in the tumors is higher than in the adjacent non-tumor tissues (signed by star). [file 1476-4598-12-164-S2.tiff]

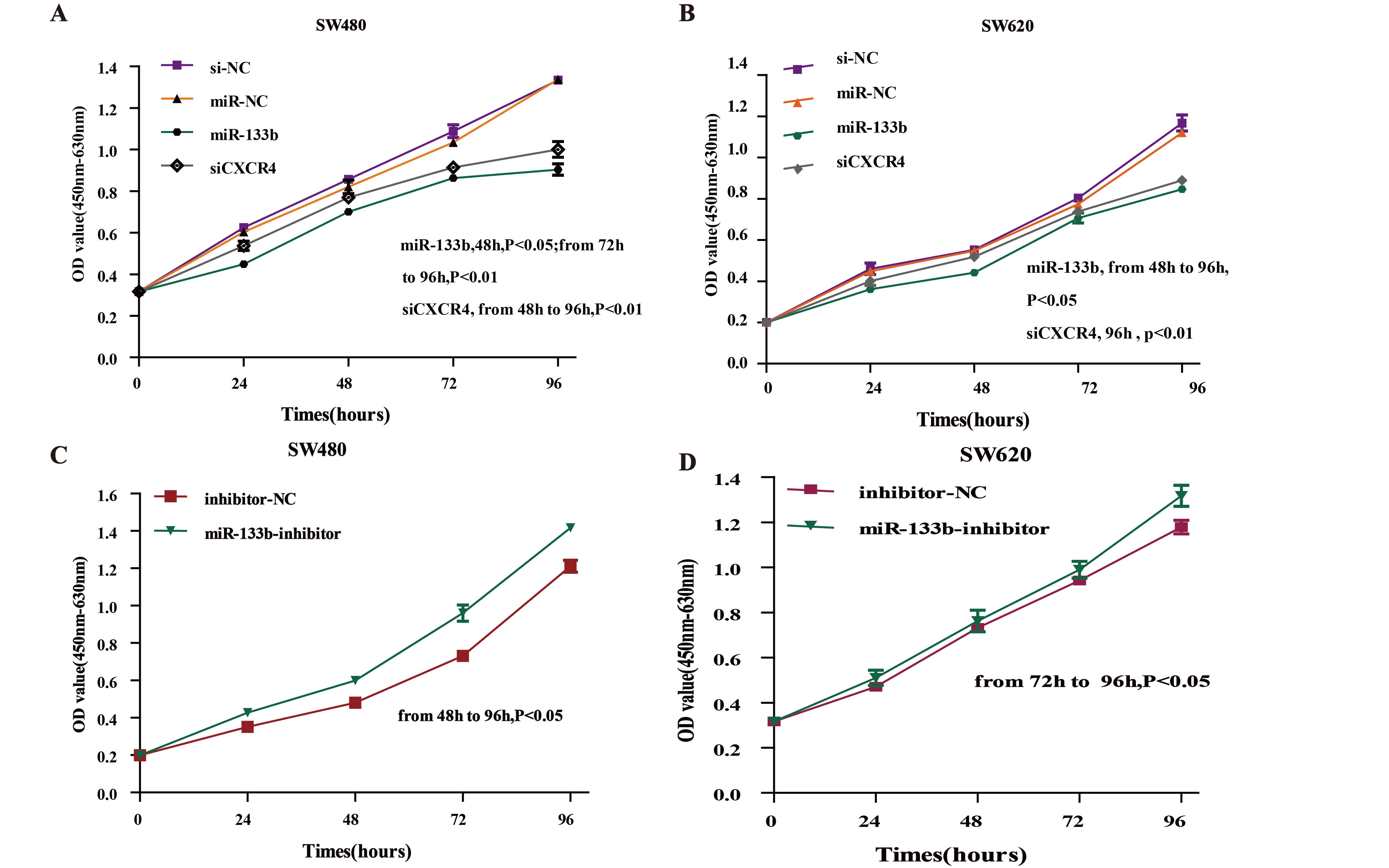

Supplement: Additional file 3: Figure S3 — The effect of miR-133b on CRC proliferation. [file 1476-4598-12-164-S3.tiff]

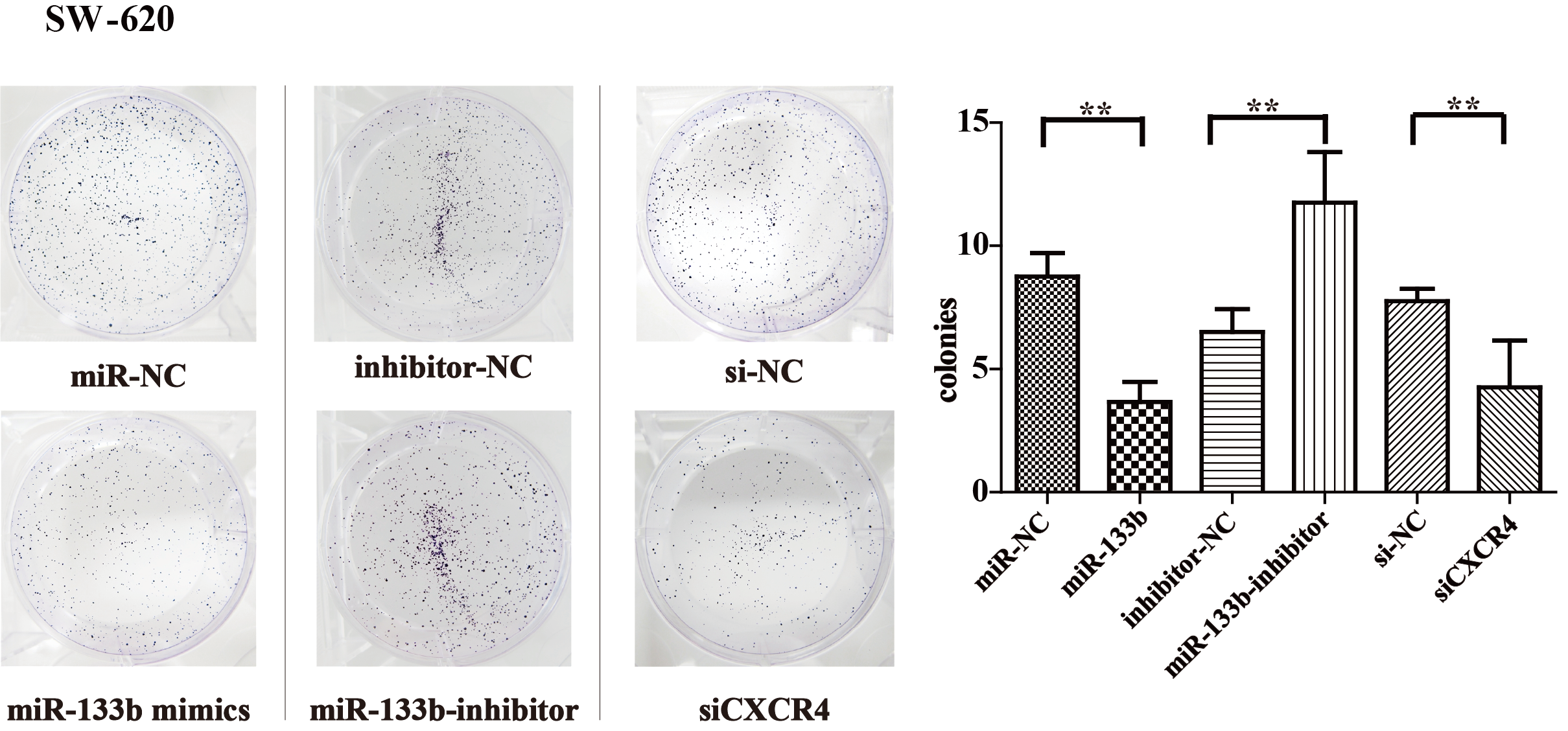

Supplement: Additional file 4: Figure S4 — Colony formation assay performed in SW-620. The number of colonies was counted under a microscope using an original magnification of 100× to adjust for the different density of each cell line. [file 1476-4598-12-164-S4.tiff]

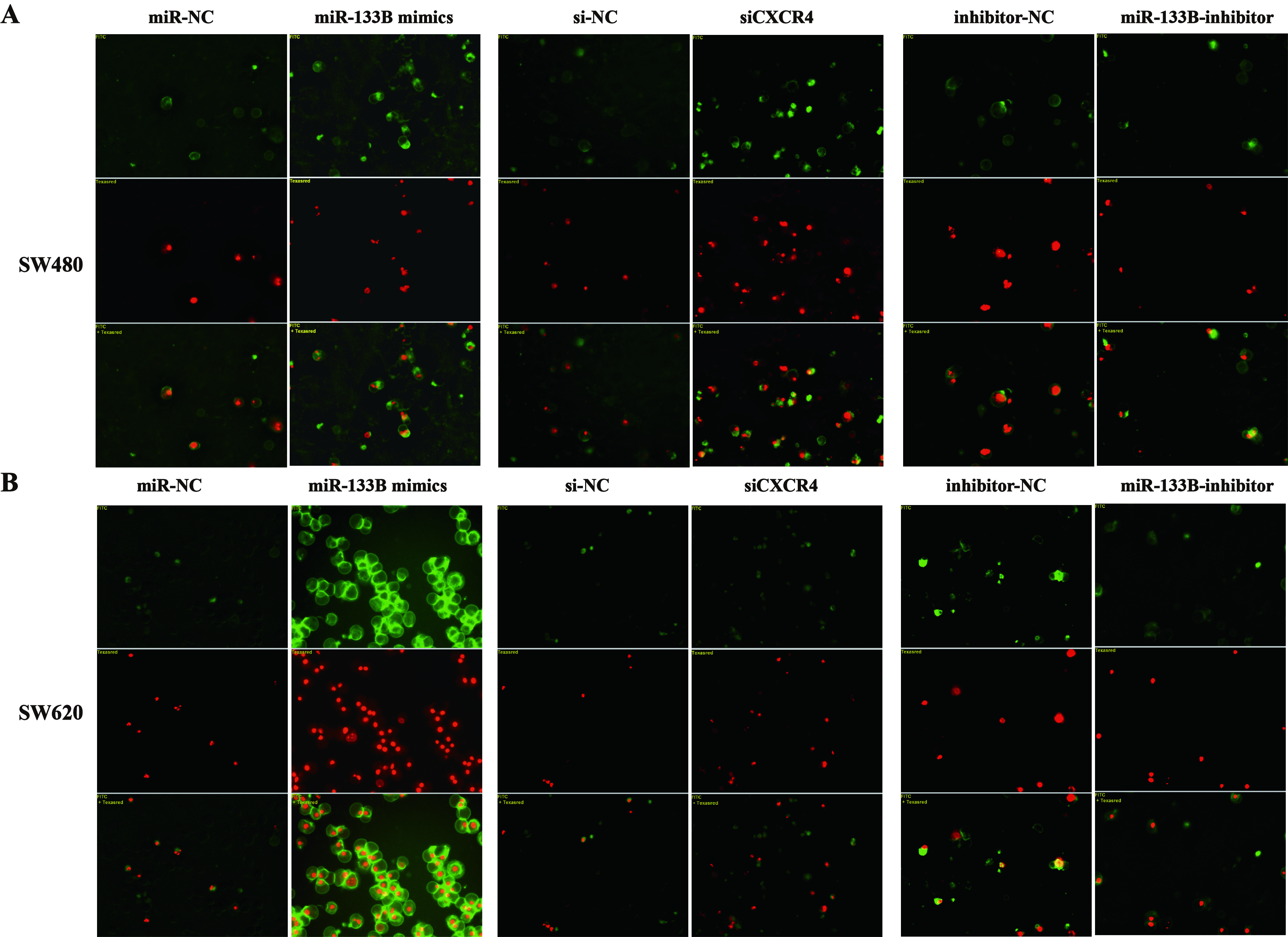

Supplement: Additional file 5: Figure S5 — (A) Observation of the apoptosis of SW-480 cells transfected with small molecules under a fluorescent microscopy. (B) Observation of the apoptosis of SW-620 cells transfected with small molecules under a microscopy. Original magnification: 200×. [file 1476-4598-12-164-S5.tiff]
