# Supplementary material for: miR-133b, a muscle-specific microRNA, is a novel prognostic marker that participates in the progression of human colorectal cancer via regulation of CXCR4 expression
Source: Mol Cancer. 2013 Dec 13;12:164. doi: 10.1186/1476-4598-12-164 (PMC3866930; doi:10.1186/1476-4598-12-164)
Supplement: Additional file 6: Table S1 — Primers and siRNA sequences used in this study. [file 1476-4598-12-164-S6.doc]

Table S1 Primers and siRNA sequences used in this study

| **Primer Name** |  | **Sequence of primer (**5'-3'**)** |
| --- | --- | --- |
| CXCR4-3-utr-59nt-wt  CXCR4-3-utr-59nt-full-mut  CXCR4-qRT  CXCR4-si-1  CXCR4-si-2 | Forward    Reverse  Forward    Reverse  Forward  Reverse  Sense  Sense | TCGAGAGTTACACATTTTTCAGATATAAAAG  ACTGACCAATATTGTACAGTTTTTATGC  GGCCGCATAAAAACTGTACAATATTGGTCAG  TCTTTTATATCTGAAAAATGTGTAACTC  TCGAGAGTTACACATTTTTCAGATATAAAAG  ACTCTGGTTTATTGTACAGTTTTTATGC  GGCCGCATAAAAACTGTACAATAAACCAG  AGTCTTTTATATCTGAAAAATGTGTAACTC  GGAGGGGATCAGTATATACA  GAAGATGATGGAGTAGATGG  UAAAAUCUUCCUGCCCACCdTdT  GGAAGCUGUUGGCUGAAAAdTdT |

**^*^** The grey font indicated the MRE(miRNA response element) on the 3’UTR of CXCR4.
